# Novel pathway of 3-hydroxyanthranilic acid formation in limazepine biosynthesis reveals evolutionary relation between phenazines and pyrrolobenzodiazepines

**DOI:** 10.1038/s41598-018-26179-w

**Published:** 2018-05-17

**Authors:** Magdalena Pavlikova, Zdenek Kamenik, Jiri Janata, Stanislav Kadlcik, Marek Kuzma, Lucie Najmanova

**Affiliations:** Institute of Microbiology of the Czech Academy of Sciences, 142 20 Prague 4, Czech Republic

## Abstract

Natural pyrrolobenzodiazepines (PBDs) form a large and structurally diverse group of antitumour microbial metabolites produced through complex pathways, which are encoded within biosynthetic gene clusters. We sequenced the gene cluster of limazepines and proposed their biosynthetic pathway based on comparison with five available gene clusters for the biosynthesis of other PBDs. Furthermore, we tested two recombinant proteins from limazepine biosynthesis, Lim5 and Lim6, with the expected substrates *in vitro*. The reactions monitored by LC-MS revealed that limazepine biosynthesis involves a new way of 3-hydroxyanthranilic acid formation, which we refer to as the chorismate/DHHA pathway and which represents an alternative to the kynurenine pathway employed for the formation of the same precursor in the biosynthesis of other PBDs. The chorismate/DHHA pathway is presumably also involved in the biosynthesis of PBD tilivalline, several natural products unrelated to PBDs, and its part is shared also with phenazine biosynthesis. The similarities between limazepine and phenazine biosynthesis indicate tight evolutionary links between these groups of compounds.

## Introduction

Pyrrolo[2,1-*c*][1,4]benzodiazepines (PBDs) bind to the minor groove of DNA, resulting in the modification or disruption of various cellular processes^1,2^. Owing to their remarkable antitumour activities, PBDs have recently been extensively studied: dozens of synthetic PBD dimers or PBD-based antibody drug conjugates were tested and the most promising candidates are being evaluated in pre-clinical tests or in phase I, II and III clinical trials^3–7^. Natural PBDs are produced predominantly by soil actinobacteria through pathways encoded within biosynthetic gene clusters (BGCs), which have been published for five PBDs (anthramycin, sibiromycin, tomaymycin, porothramycin and tilivalline)^8–12^.

**Figure 1:**
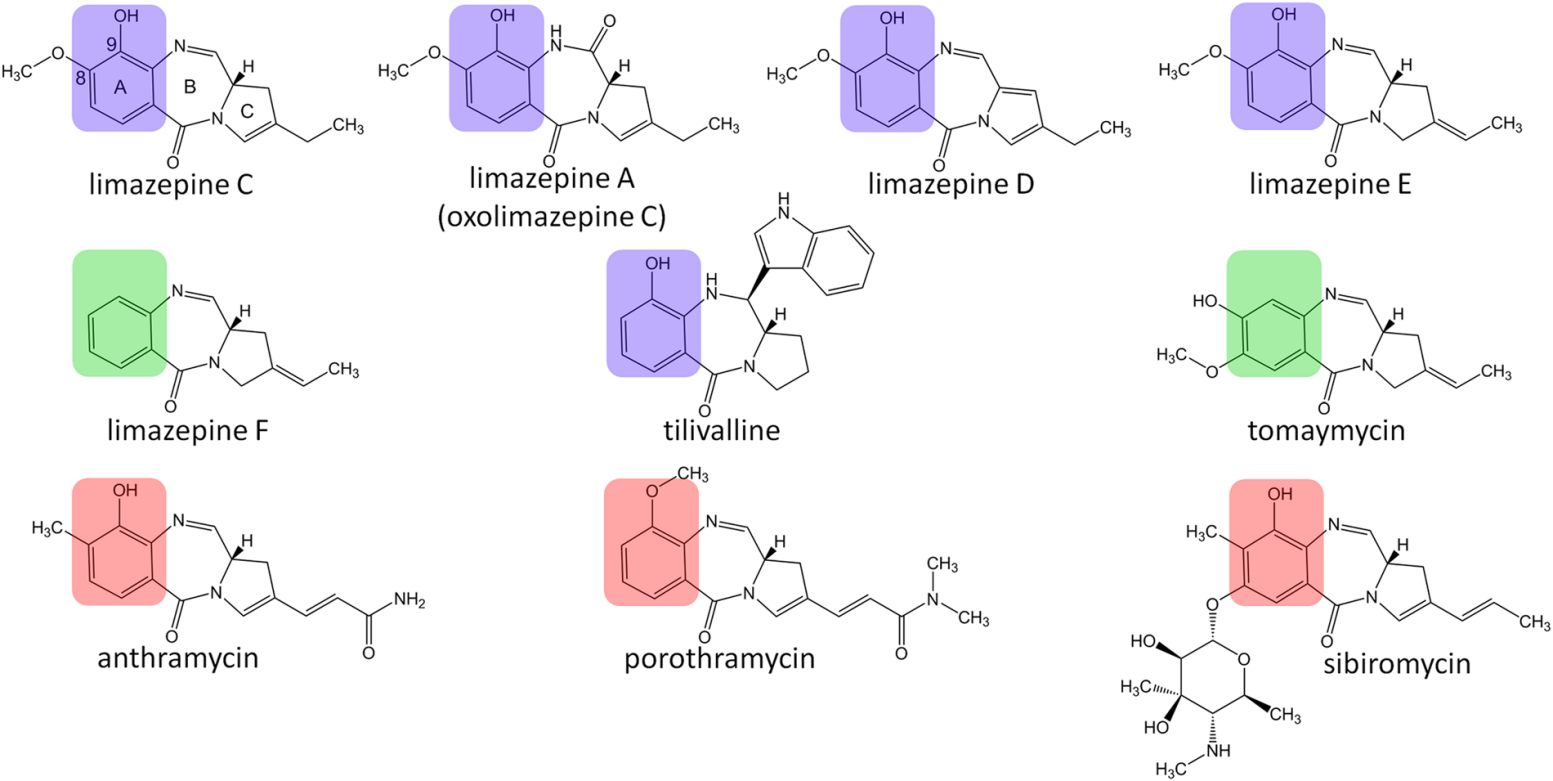
Structures of PBDs with previously published or here reported BGCs. Anthranilate moieties are highlighted in colours according to the biosynthetic strategy of their formation: chorismate/anthranilate pathway (not hydroxylated at C-9; highlighted in green), kynurenine pathway (hydroxylated at C-9; highlighted in red) or here elucidated chorismate/DHHA pathway (hydroxylated at C-9; highlighted in violet).

**Figure 2:**
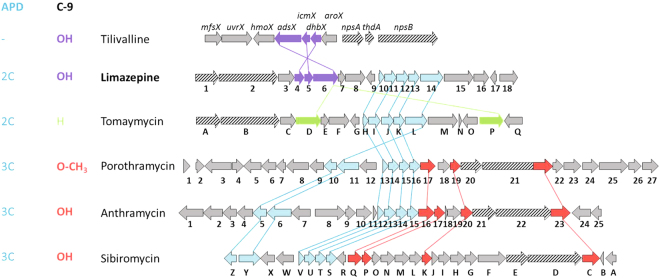
Comparison of biosynthetic gene clusters of PBDs. The genes are marked with the respective numbers or capital letters (for example 1 stands for *lim1* in limazepine BGC, *por1* in porothramycin BGC or *orf1* in anthramycin BGC while A stands for *tomA* in tomaymycin BGC and *sibA* in sibiromycin BGC). Genes coding for APD precursor biosynthesis are in cyan; genes coding the biosynthesis of anthranilic acid derivatives are highlighted according to the colours in Fig. 1 (red for kynurenine pathway, violet for chorismate/DHHA pathway, green for chorismate/anthranilate pathway); genes assigned to NRPS are striped. Sequentially homologous genes relevant to the discussed biosynthetic steps are linked. The presence/type of APD precursor incorporated into the final PBD and the tailoring at C-9 are indicated at the left.

The tricyclic PBD molecule is biosynthesized from two precursors, anthranilic acid or its derivative (ring A) and L-proline or its derivative (ring C, Fig. 1). Their condensation, which is catalysed by a nonribosomal peptide synthetase (NRPS), is followed by a spontaneous internal cyclisation of the dipeptide resulting in the diazepine ring formation (ring B). L-proline derivatives and/or anthranilic acid derivatives formed in specialized pathways of secondary metabolism are commonly incorporated into the majority of PBDs. For instance, evolutionary more advanced 4-alkyl-L-proline derivatives (APDs) are incorporated into PBDs instead of L-proline. APDs are synthesized from L-tyrosine in a biosynthetic pathway catalysed by five (for APDs with a two-carbon alkyl side chain; i.e. 2C APDs) or six enzymes (for APDs with a three-carbon alkyl side chain; i.e. 3C APDs)^13,14^. This corresponds to the presence of a five- or six-membered APD sub-cluster in the BGCs of PBDs^8–11^ (Fig. 2). APDs are incorporated not only into PBDs, but also two other structurally and functionally distinct natural products, bacterial signalling molecule hormaomycin^15^ and lincosamide antibiotic lincomycin^16^. The anthranilic acid-derived moieties of PBDs have been known to be formed through two distinct biosynthetic machineries, kynurenine and chorismate/anthranilate pathways. The kynurenine pathway is applied to form 3-hydroxyanthranilic acid derivatives and it is accordingly encoded within the BGCs of anthramycin, sibiromycin and porothramycin^8,10,11,17^, in which this hydroxyl is present at the C-9 position of the resulting PBD skeleton. The chorismate/anthranilate pathway is employed to form C-3 unsubstituted anthranilic acid precursors and it is accordingly encoded within the BGC of C-9 unsubstituted PBD tomaymycin^2,9,18^.

In this paper, we present the sequence of limazepine BGC, i.e. the sixth gene cluster of PBDs. Based on its sequence analysis and functional elucidation of two limazepine biosynthetic proteins, we show that 3-hydroxyanthranilic acid precursors of PBDs can be, apart from kynurenine pathway, biosynthesized also through a novel pathway, which we refer to as chorismate/DHHA pathway (DHHA stands for *trans*-2,3-dihydro-3-hydroxyanthranilic acid). Furthermore, part of the chorismate/DHHA pathway is identical to the initial steps of the biosynthesis of phenazines, which allowed us to document the evolutionary linkage between phenazines and PBDs.

## Materials and Methods

### DNA techniques, genome sequencing and analysis

Chromosomal DNA was isolated from *Streptomyces* sp. ICBB 8177^19,20^ according to method described by Hopwood *et al*.^21^ and modified by Vachalova *et al*.^22^. The genomic library was prepared using TruSeq^®^ DNA PCR-Free Library Preparation Kit (Illumina) and, sequenced on the Illumina MiSeq platform (GeneTiCA, Czech Republic). The Velvet 1.2.10, Bowtie 2 and Khmer programs were utilized to perform the *de novo* assembly of the sequencing data.

The putative limazepine BGC was identified using antiSMASH^23^. The ORFs were predicted more precisely using FgenesB^24^ and Glimmer (V3.02)^25^ and manually edited based on homology with already sequenced PBD gene clusters or other closely related genes. The BlastX (http://blast.ncbi.nlm.nih.gov/Blast.cgi) was used for prediction of putative functions of encoded proteins^26^.

Sequence of the *Streptomyces* sp. ICBB 8177 genome and limazepine BGC was deposited in the GenBank under the accession number NSKH00000000 and KT381463, respectively.

### Production of recombinant Lim6 and Lim5

Genes *lim6* and *lim5* were PCR amplified using primer pairs listed in Table 1. The PCR products were inserted into pET28b vector (Novagen) via NdeI and EcoRI restriction sites and the resulting constructs were used to produce soluble N-terminally His_6_-tagged proteins in *E. coli* BL21(DE3) (Novagen). Both proteins Lim6 and Lim5 were co-expressed with GroES and GroEL chaperonins. Overexpression was induced by 0.4 mM isopropyl-β-D-thiogalactopyranoside. After induction, the cells were grown for 20 hours at 17 °C, harvested by centrifugation (4200 rpm, 20 min, 4 °C) and stored frozen at −20 °C. The cells were disrupted by ultrasonic homogenization in TS-8 buffer (20 mM Tris, 100 mM NaCl, pH 8.0). Lim6 and Lim5 were purified using HiTrap™ Chelating HP Columns (GE Healthcare) equilibrated with TS-8 buffer. The proteins were eluted by TS-8 buffer with 250 mM imidazole, exchanged to TS-8 using 30-kDa Amicon cartridges (Millipore) and immediately used for enzymatic assays.

**Table 1:** Primers for *lim6* and *lim5* genes amplification.

Name	Sequence
lim6_forward	ATATCATATGACCGGCGCGCCGTA
lim6_reverse	ATATAGAATTCTAGCGGCTGCCATGGGC
lim5_forward	ATATCATATGACCGCGACCACCGCC
lim5_reverse	ATATAGAATTCTCATGTCGTTCCCCCGTCG

Restriction sites are underlined.

### Enzymatic assays

To confirm the proposed aminodesoxyisochorismate synthase activity of Lim6, the following *in vitro* assay was used. The reaction mixture of 100 mM Tris buffer (pH 8.0), 5 mM chorismic acid (barium salt, from *Enterobacter aerogenes*, Sigma-Aldrich), 20 mM L-glutamine, 5 mM MgCl_2_ and purified enzyme Lim6 in a total volume of 100 µL was incubated for 1 h at 30 °C. To confirm the proposed subsequent transformation of 2-amino-2-desoxyisochorismic acid (ADIC) to *trans*-2,3-dihydro-3-hydroxyanthranilic acid (DHHA) by Lim5, the reaction with Lim6 was prepared and after 1 h of incubation, Lim5 was added and the reaction was incubated for additional 1 h under the same conditions. The activity of Lim5 was also tested with chorismic acid as a substrate in a reaction containing 100 mM Tris buffer (pH 8.0), 20 mM chorismic acid and Lim5 in a total volume of 100 µL, incubated for 1 h at 30 °C. To elucidate whether the order of Lim6 and Lim5 reactions for conversion of chorismic acid to DHHA is strict, we extracted *trans*-3,4-dihydro-3,4-dihydroxybenzoic acid from the reaction of chorismic acid with Lim5 as described below and used it as a substrate of Lim6 (except of the tested substrate, the reaction conditions were identical to the reaction of chorismic acid with Lim6). For all tested reactions, negative controls were prepared as the same reaction mixtures with the TS-8 buffer instead of the protein solutions. The assays were terminated by adding 4 µL formic acid, were centrifuged (13000 rpm, 20 min, 4 °C) and analysed by LC-MS.

### LC-MS analysis

LC-MS analyses were performed on the Acquity UPLC system with LCT premier XE time-of-flight mass spectrometer (Waters, USA). Five µL of sample were loaded onto the Acquity UPLC CSH C18 LC column (50 mm × 2.1 mm I.D., particle size 1.7 μm, Waters) kept at 40 °C and eluted with a two-component mobile phase, A and B, consisting of 0.1% formic acid (98–100%, Merck, Germany) and acetonitrile (LC-MS grade, Biosolve, Netherlands), respectively. The analyses were performed under a linear gradient program (min/%B) 0/5, 1.5/5, 12.5/58 followed by a 1.5-min column clean-up (100% B) and 1.5-min equilibration (5% B), at the flow rate of 0.4 mL min^−1^. The mass spectrometer operated in the “W” mode with capillary voltage set at +/−2800 V, cone voltage +/−40 V, desolvation gas temperature, 350 °C; ion source block temperature, 120 °C; cone gas flow, 50 Lh^−1^; desolvation gas flow, 800 Lh^−1^; scan time of 0.15 s; inter-scan delay of 0.01 s; inter-scan delay between polarity switch, 0.1 s. The mass accuracy was kept below 5 ppm using lock spray technology with leucine enkephalin as the reference compound (2 ng μL^−1^, 5 μL min^−1^). Chromatograms were extracted for [M + H]^+^ or [M − H]^−^ ions with the tolerance window of 0.05 Da. The data were processed by MassLynx V4.1 (Waters).

### Extraction of reaction products

The enzymatic reactions were scaled up to the volume of 3.6 mL and after the reaction termination with 150 uL formic acid, each reaction was loaded on an Oasis MCX (1 g) solid phase extraction cartridge (Waters, USA), pre-conditioned with 30 mL methanol and 30 mL 2% formic acid in water. The column flow-through contained *trans*-3,4-dihydro-3,4-dihydroxybenzoic acid if present in the reactions. The column was then washed with 30 mL 2% formic acid in water and 15 mL methanol and DHHA or ADIC (according to the reaction composition) was eluted with 15 mL methanol:ammonium hydroxide 95:5 (v/v). The extracts were evaporated to dryness and used for further enzymatic assays or NMR experiments.

### Data availability

The datasets generated during and/or analysed during the current study are available in the GenBank repository under accession numbers NSKH00000000 and KT381463.

## Results and Discussion

### Biosynthetic gene cluster of limazepines

*Streptomyces* sp. ICBB 8177 was previously shown to produce limazepine PBDs, specifically limazepines C, D, E, and F and the C-11-oxo-derivative of limazepine C named limazepine A^19^. We acquired the *Streptomyces* ICBB 8177 genome sequence by MiSeq technology and the assembly of raw data (sequence coverage 124x) provided us with a draft genome sequence of 6 331 712 bp in 28 contigs (Accession number NSKH00000000). The 25568 bp long limazepine BGC (Fig. 2) was detected using AntiSMASH^23^ and deposited in GenBank under the Accession number KT381463. Eighteen open reading frames (ORFs) named *lim1* – *lim18* were identified within the BGC and their products were assigned to limazepine biosynthesis according to BlastX analysis (Table 2).

**Table 2:** Analysis of limazepine biosynthetic gene cluster.

Protein	Homologous proteins (from PBDs and phenazines)					Phenazines	Identity/similarity to homologue with*	Function of limazepine biosynthetic protein
Limazepine	Tomaymycin	Anthramycin	Porothramycin	Sibiromycin	Tilivalline			
**outside biosynthetic gene cluster**								
ORF1	—	—	—	—	—	—	49/60	chromosome segregation protein Spo0J, highest homology to: WP_020636716.1* (primary metabolism)^C^
**PBD assembly**								
Lim1	TomA^18^*	ORF21	POR20	SibE^17^	NpsA, ThdA	—	50/62	NRPS (anthranilate precursor activation)^A^
Lim2	TomB^18^*	ORF22	POR21	SibD	NpsB	—	50/61	NRPS (APD precursor activation and condensation)^A^
**biosynthesis of anthranilate moiety**								
Lim3	TomC*	—	—	—	AroX	PhzC	62/73	DAHP synthase^B^
Lim4	—	—	—	—	DhbX*	—	50/59	DHHA oxidoreductase^B^
Lim5	—	—	—	—	IcmX*	PhzD	53/65	isochorismatase (pyruvate removal from ADIC)^C^
Lim6	TomD* TomP	—	—	—	AdsX	PhzE	56/65	ADIC synthase^C^
Lim7	TomE*	—	—	—	—	—	61/71	oxidoreductase (hydroxylation)^B^
Lim8	TomF*	—	—	—	—	—	83/90	monooxygenase (hydroxylation)^B^
Lim9	TomG*	—	—	—	—	—	62/73	methyltransferase (*O-*methylation)^B^
**biosynthesis of APD**								
Lim10	TomH*	ORF12^48^	POR13	SibV^48^	—	—	65/73	L-DOPA-2,3-dioxygenase (oxidative cleavage of L-DOPA)^A^
Lim11	TomI	ORF13^49^	POR14	SibU*	—	—	49/62	tyrosine hydroxylase (synthesis of DOPA)^A^
Lim12	TomJ*	ORF14	POR15	SibT	—	—	67/78	F-420 dependent reductase (double bond reduction)^D^
Lim13	TomK*	ORF15	POR16	SibS	—	PhzF	47/56	Isomerase (double bond isomeration)^D^
Lim14	TomL*	ORF6^50^	POR11	SibY	—	—	66/76	ƴ- glutamyltransferase-like hydrolase (C-C bond cleavage)^A^
**resistance/regulation/unknown function**								
Lim15	TomM	ORF8*	POR3	SibF	UvrX	—	72/84	excinuclease ABC subunit A (resistance)^B^
Lim16	—	—	—	—	—	—	96/98	LLM class flavin-dependent oxidoreductase, highest homology to: WP_051872335.1* (unknown function)^B^
Lim17	—	—	—	—	—	—	61/76	MarR family DNA-binding transcriptional regulator, highest homology to: WP_086678485.1* (regulation)^B^
Lim18	TomQ*	ORF24	POR5	—	—	—	67/79	amine oxidase (unknown function)^B^
**outside biosynthetic gene cluster**								
ORF2	—	—	—	—	—	—	70/79	cation:proton antiporter, highest homology to: WP_030262790.1* (primary metabolism)^B^

^*^Indicates the closest homologue of the respective Lim protein.

^A^Proposed based on elucidation of a homologue from PBD biosynthesis (reference included).

^B^Proposed based on sequence homology.

^C^Confirmed *in vitro* in this study.

^D^Proposed based on indirect *in vivo* studies of lincomycin biosynthesis, which shares APD biosynthesis with PBDs^14^.

*Orf1* upstream *lim1* and *orf2* downstream *lim18* do not exhibit any homology to already described ORFs of any PBD biosynthetic gene cluster. According to BlastX and Conserved domain search tool at NCBI^27^, *orf1* encodes a protein of the Spo0J superfamily, which contains a ParB-like nuclease domain. These proteins are known to participate in the cell division and chromosome partitioning. *Orf2* encodes a protein homologous to cation:proton antiporters, which serve as the key transporters in maintaining the pH of actively metabolising cells. Products of *orf1* and *orf2* presumably belong to primary metabolism, not to limazepine biosynthesis. Therefore, we consider *lim1* and *lim18* as the boundary ORFs of the limazepine BGC.

### Limazepine biosynthesis–elucidation of novel chorismate/DHHA pathway

**Figure 3:**
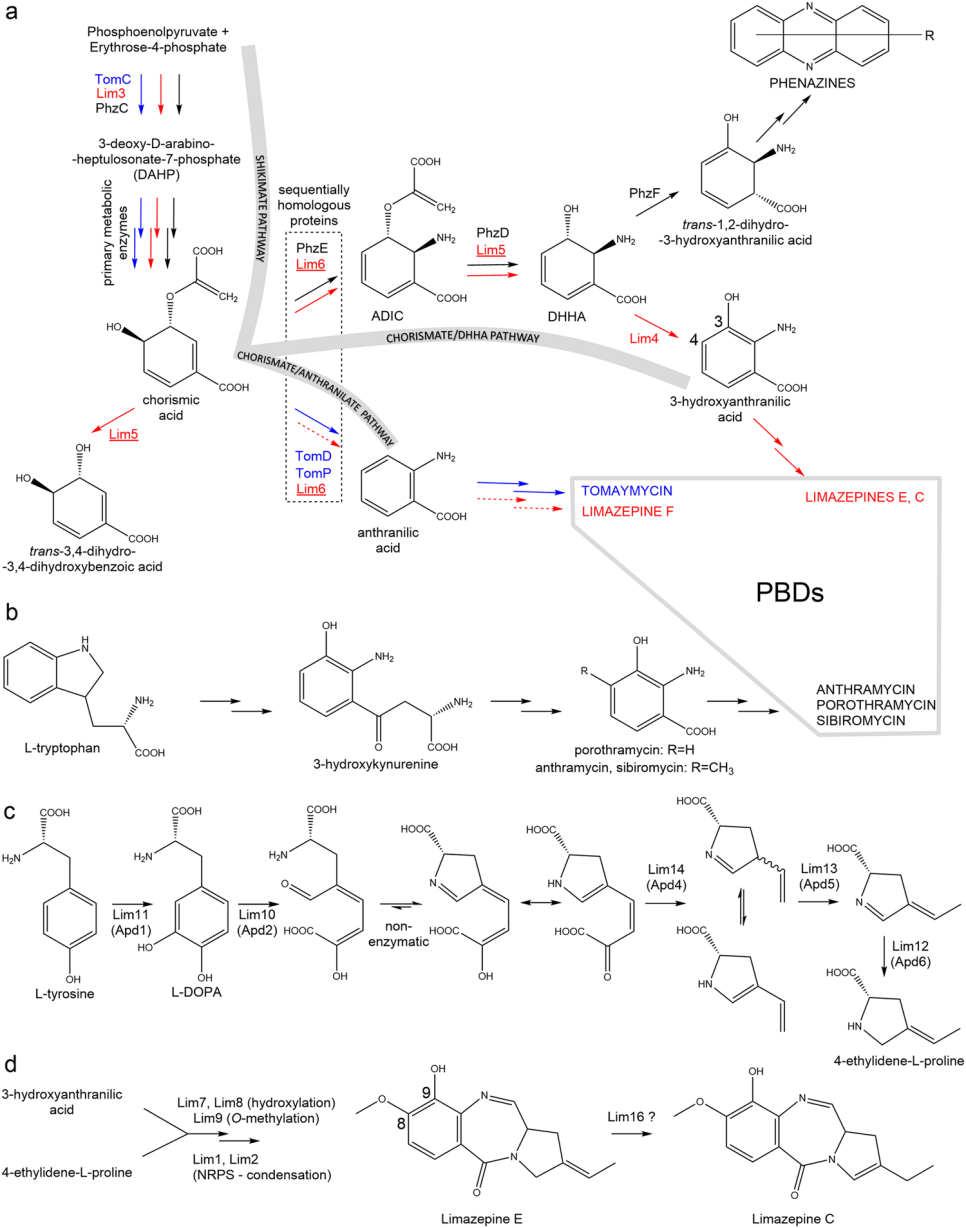
Biosynthesis of PBDs. (**a**) Chorismate pathways: chorismate/anthranilate pathway for the biosynthesis of anthranilic acid precursors incorporated into tomaymycin (blue) and limazepine F (red) and here elucidated chorismate/DHHA pathway for the biosynthesis of 3-hydroxyanthranilic acid precursors incorporated into limazepines E and C (red). Biosynthetic steps up to DHHA are shared with the biosynthesis of phenazines (black). Proteins elucidated in this study are underlined; the minor biosynthetic stream is indicated by dashed arrows; (**b**) kynurenine pathway for the biosynthesis of 3-hydroxyanthranilic acid precursors incorporated into anthramycin, porothramycin and sibiromycin; (**c**) limazepine APD biosynthetic pathway^14^; (**d**) proposed assembly of limazepines.

Fourteen out of the 18 genes of limazepine BGC have their counterparts in the BGC of tomaymycin^9^ (Table 2), indicating even higher biosynthetic similarity than the respective PBD structures suggest. Specifically, we did not identify the set of genes coding for the kynurenine pathway (Fig. 3b) leading to the formation of anthranilic acid precursors hydroxylated at C-3. We presumed employment of this pathway because limazepines (in contrast to tomaymycin) contain the corresponding hydroxyl group at C-9 of the anthranilate moiety. Instead, we identified genes *lim3* and *lim6*, encoding proteins homologous to those from the shikimate and chorismate/anthranilate pathway, respectively, of tomaymycin biosynthesis where they are involved in the formation of C-3 unsubstituted anthranilic acid derivative (Fig. 3a)^9^. The chorismate/anthranilate pathway follows the seven reaction steps of the primary metabolic shikimate pathway, which starts from 3-desoxy-D-arabinoheptulosonate 7-phosphate (DAHP) formation catalysed by DAHP synthase. While homologues of this protein are encoded within both tomaymycin (*tomC*)^9^ and limazepine (*lim3*) BGCs, the remaining six steps of shikimate pathway leading to chorismic acid are probably carried out by primary metabolic proteins as it was described e.g. in the biosynthesis of phenazines^28^. The additional copy of the DAHP synthase gene is probably present in the limazepine and tomaymycin BGCs in order to overcome the regulatory bottleneck of the primary metabolic shikimate pathway to ensure sufficient chorismic acid pool for the respective secondary metabolite biosynthesis. In tomaymycin biosynthesis, chorismic acid is proposed to be converted into anthranilic acid by a pair of mutually homologous putative anthranilate synthases, TomD and TomP (Fig. 3)^9^. Anthranilate synthases of primary metabolism usually initiate tryptophan biosynthesis. They convert chorismic acid to anthranilic acid in two steps: The first step involves the transfer of ammonia from glutamine to chorismate. At the same time, the chorismate hydroxyl group at C-4 is lost, affording 2-amino-2-desoxyisochorismic acid (ADIC). The second step utilizes an ADIC lyase activity of the anthranilate synthase to remove the pyruvate group (and a proton) at C-3 of ADIC, releasing anthranilic acid^29^. A gene coding for a protein homologous to anthranilate synthase, Lim 6, has also been identified in the limazepine BGC. Therefore, it could be expected to transform chorismic acid directly to anthranilic acid, as it occurs in the biosynthesis of tomaymycin, but this would contradict the presence of C9 hydroxyl in the final structure of most limazepine derivatives. However, Lim6 is also homologous to PhzE from the biosynthesis of phenazines. PhzE, closely related to anthranilate synthases, catalyses the transformation of the chorismic acid to ADIC. In contrast to anthranilate synthases, PhzE is, despite to very similar active sites, incapable of catalysing pyruvate elimination from ADIC to yield anthranilic acid. In the protein sequences, there is no indication of why anthranilate synthase further converts ADIC to anthranilate whereas PhzE does not^30^. According to Qi-Ang *et al*.^31^, the reason probably lies in the mechanism of the substrate release. In the reactions catalysed by PhzE-like proteins, it is possible that the pyruvate is not eliminated due to a reduced residence time of ADIC in the catalytic site and/or due to a different release path. It has also been speculated that different release mechanisms are a consequence of different quaternary structures^31^.

We hypothesized that the function of Lim6 is identical to that of PhzE and its reaction product is ADIC. For further ADIC conversion to the desired 3-hydroxyanthranilic acid, Lim5 and Lim4 would be suitable candidates. Lim5 is homologous to PhzD from phenazine biosynthesis, which was shown to transform ADIC to DHHA. Lim 4 is homologous to 2,3-dihydro-2,3-dihydroxybenzoate dehydrogenases and could thus be responsible for the remaining oxidation of DHHA to 3-hydroxyanthranillic acid.

**Figure 4:**
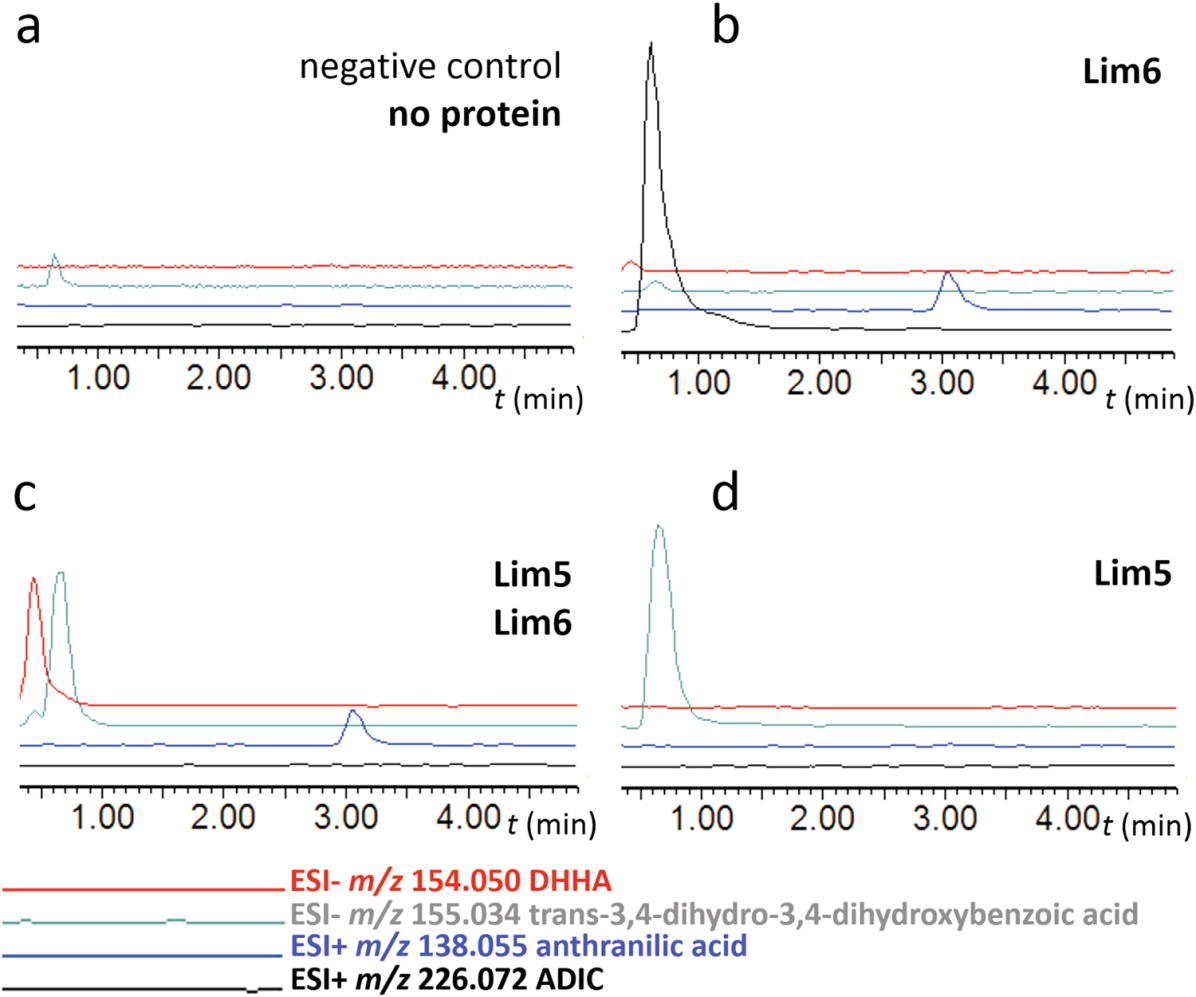
Transformation of chorismic acid to DHHA; (**a**) negative control; (**b**) conversion of chorismic acid into ADIC and anthranilic acid by Lim6; (**c**) conversion of chorismic acid into DHHA by Lim6 and Lim5; (**d**) conversion of chorismic acid into *trans*-3,4-dihydro-3,4-dihydroxybenzoic acid by Lim5.

To confirm the hypothesis of 3-hydroxyanthranilic acid formation through the new type of chorismate pathway, which we refer to as chorismate/DHHA pathway, we heterologously produced and purified Lim5 and Lim6 (alignments with their homologues are depicted in Supplementary material Fig. 1A,B, respectively) and tested them *in vitro* with the expected substrates. First, we proved that the predominant product of the reaction of Lim6 with chorismic acid is ADIC (Fig. 4b; for NMR elucidation see Supplementary material Table 1), confirming that the function of Lim6 is identical to that of PhzE and not to TomD/TomP. Unexpectedly, we also detected anthranilic acid as a minor reaction product, showing that Lim6 acts to a very low extent also as anthranilate synthase. To elucidate the subsequent course of reactions, we incubated chorismic acid with both Lim6 and Lim5. In this reaction, we still detected anthranilic acid as the minor Lim6 product. By contrast, ADIC was depleted and a new product corresponding to DHHA was detected (Fig. 4c; for NMR elucidation see Supplementary material Table 2). Furthermore, we revealed that chorismic acid can be converted also by Lim5 and we detected *trans*-3,4-dihydro-3,4-dihydroxybenzoic acid as the corresponding product (Fig. 4c,d). It documents that Lim5 can remove the acrylate not only from ADIC to afford DHHA, but also from chorismic acid to afford *trans*-3,4-dihydro-3,4-dihydroxybenzoic acid. This finding is in accordance with previously published data on a homologous protein, PhzD, which preferentially utilizes ADIC (Fig. 3a), but can also use chorismic acid as a substrate^32^. In addition, we tested whether Lim6 can convert *trans*-3,4-dihydro-3,4-dihydroxybenzoic acid to DHHA and we obtained a negative result (Supplementary material Fig. 2). It confirms that the order of reactions catalysed by Lim6 and subsequently by Lim5 to convert chorismic acid into DHHA is strict. These experiments suggest that the *in vivo* system ensures to process chorismic acid by Lim6 and not Lim5 in order to lead the pathway towards 3-hydroxyanthranilic acid.

The subsequent course of limazepine assembly (depicted in Fig. 3d) can be inferred from the recently elucidated biosynthesis of tomaymycin^18^. 3-Hydroxyanthranilic acid presumably enters the NRPS-directed condensation reaction encoded by *lim1* and *lim2* genes. While 3-hydroxyanthranilic acid is bound to the carrier protein domain of Lim1, the additional substitutions catalysed by Lim7 and Lim8 (hydroxylation of C-8) and Lim9 (*O*-methylation of the C-8 hydroxyl) probably occur. Biosynthesis of the other condensing partner, 4-ethylidene-L-proline, is presumably encoded by a set of five genes, *lim10* – *lim14* (Fig. 3c), which are homologous to the genes of the APD sub-cluster encoding biosynthesis of APD precursors in other PBDs, lincomycin, and hormaomycin. The resulting limazepine formed through the presented pathway specifically corresponds to limazepine E, but formation of its derivatives can be inferred too. We assume that limazepine F is formed when anthranilic acid (as a product of primary metabolism or as a minor product of Lim6 reaction) is incorporated instead of 3-hydroxyanthranilic acid. In limazepine C, the APD moiety possesses a single endocyclic double bond. We propose that it is formed as a post-condensation modification of limazepine E by the APD double bond shift. The putative oxidoreductase Lim16, which does not have any homologue in the biosynthesis of other PBDs, could participate in this reaction. As follows from earlier observations^19^, limazepine D probably arises spontaneously as a more stable derivative of limazepine C and we propose that there is no corresponding activity encoded within the gene cluster. Formation of C-11-oxo derivative of limazepine C, which was described for example also for tomaymycin^9^ or RK-1441A^33^ and which we detected for other PBDs (data not shown) is unclear because the comparison of relevant biosynthetic gene clusters does not offer any candidates for this oxidation step and it is thus possible that also this process does not require an activity encoded within the gene cluster.

### Evolutionary links between limazepines and phenazines

**Figure 5:**
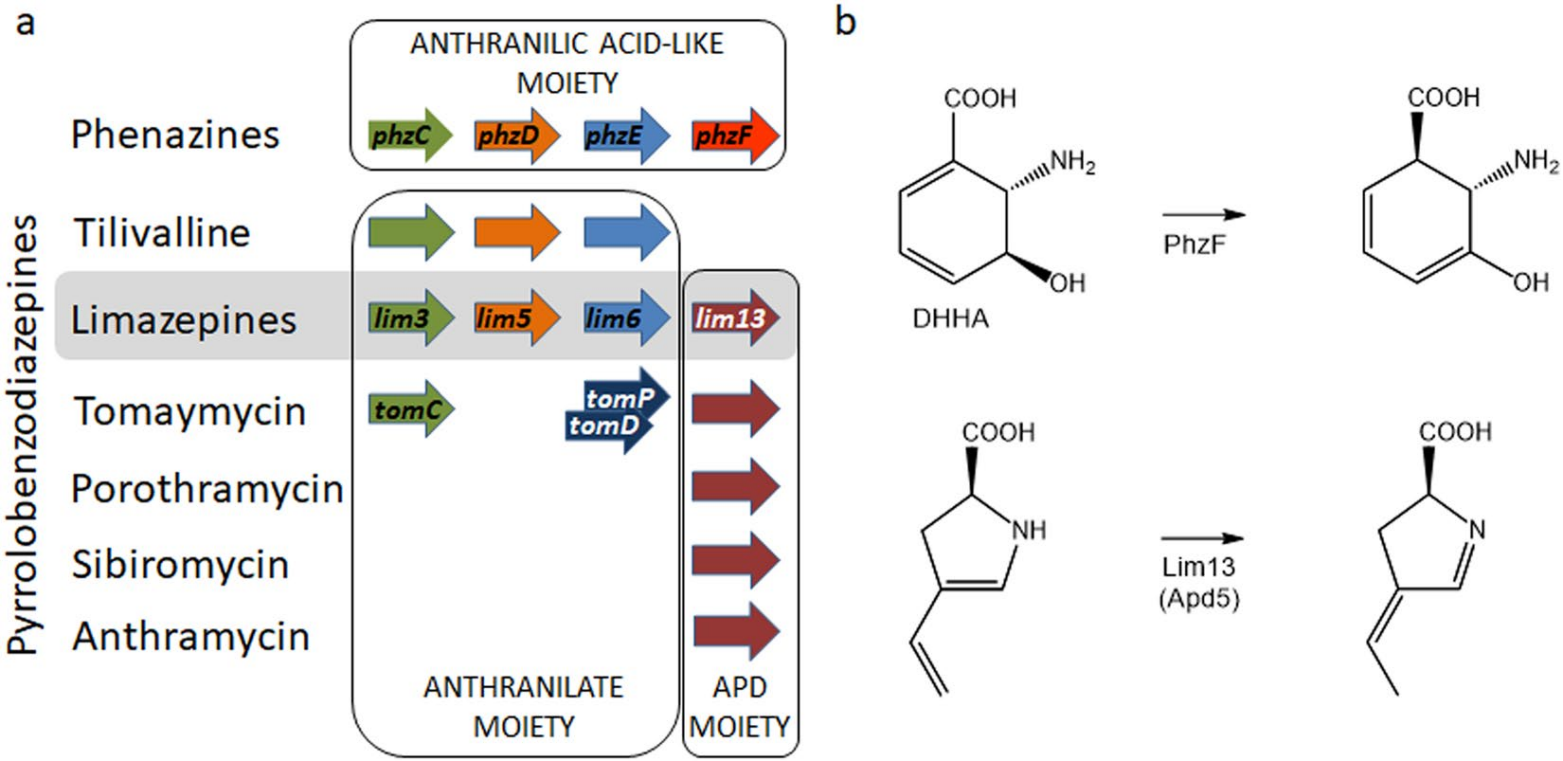
Sequential and functional homologues in phenazine vs. PBD biosyntheses. (**a**) presence of homologues of four phenazine biosynthetic genes in BGCs of PBDs; genes encoding sequential homologues are in columns and genes encoding also functional homologues are of the same colour shade; limazepines are highlighted in grey as the only BGC of PBDs encoding sequential homologues of all four phenazine biosynthetic genes; (**b**) comparison of substrates and reactions catalysed by PhzF and putatively catalysed by the PhzF sequential homologue, Lim13 (Apd5).

Part of the chorismate/DHHA pathway of 3-hydroxyanthranilic acid-precursor of limazepines is shared with the biosynthesis of phenazines^30^ (Table 2, Fig. 3a), which are distinct from PBDs in terms of their structure as well as more frequent natural occurrence. Specifically, both biosynthetic pathways employ homologous enzymes (PhzC/Lim3, PhzE/Lim6, PhzD/Lim5) to produce DHHA, which is converted to 3-hydroxyanthranilic acid by putative oxidoreductase Lim4 in limazepine biosynthesis but to *trans*-1,2-dihydro-3-hydroxyanthranilic acid by PhzF isomerase in phenazine biosynthesis^34,35^. Even though the common DHHA intermediate is processed differently in limazepine and phenazine biosyntheses (Fig. 3a), a homologue of PhzF isomerase is encoded also within the BGC of limazepines. However, this PhzF sequential homologue, Lim 13, is a putative isomerase involved in the biosynthesis of APD^14^, the other precursor of limazepines (Fig. 5).

We consider that the involvement of part of the chorismate/DHHA pathway in the otherwise unrelated limazepine and phenazine biosyntheses together with obvious evolutionary relationship of Lim13 and PhzF may not be coincidental. Therefore, we presume that biosynthetic pathways of limazepines and phenazines directly encountered during their evolution, for which we elaborate the evidences in more detail below.

PhzF and APD biosynthetic protein Lim13 and its homologues from other APD pathways (thereinafter referred to as Apd5 according to the order of the catalysed reaction in APD biosynthesis; see Fig. 3c and a review)^36^ belong to the protein family of isomerases together with primary metabolic proline racemases^37^ and diaminopimelate (DAP) epimerases^38^. The mutual sequence homology of Apd5 and PhzF (up to 30% of identity); however, significantly exceeds homologies of these proteins to the above mentioned primary metabolic isomerases (13 to 16% of identity). This finding corresponds with the phylogenetic analysis (Supplementary material Fig. 3), which documents evolutionary relationship of PhzF and Apd5 proteins from various sources. Further, proline racemases and DAP epimerases typically possess two conserved catalytic cysteine residues^39^. In contrast, PhzF isomerases do not employ this pair of cysteine residues, but a catalytic glutamate^40^, which is conserved also in all Apd5 putative isomerases (Supplementary material Fig. 1c). Therefore, we assume that an analogous mechanism of reaction takes place in the case of DHHA isomeration by PhzF as well as unsaturated 4-alkyl-L-proline derivative isomeration by Apd5^14^ (Fig. 5).

It would be too speculative to draw a specific hypothesis about the PhzF- and Apd5-related evolutionary events and their direction. However, we propose that among all natural compounds with an APD moiety, the evolutionary origin of Apd5 lies among PBDs. That is because Apd5 is encoded within limazepines and all four additional characterized (Fig. 5) as well as all 19 hypothetical^36^ BGCs of PBDs with an APD moiety. This obligatory presence of Apd5 has a functional explanation: isomerization reaction putatively catalysed by Apd5 results in a planar conformation of the side chain of APD precursors and consequently in the planar shape of the final PBD molecules, which thus fits perfectly within the target structure, i.e. the DNA minor groove^36,41^. In contrast to PDBs, Apd5 is rare in the biosynthesis of other complex natural compounds, which also incorporate an APD precursors, but are otherwise unrelated to PBDs (*apd5* was found only in four out of 19 such BGCs), suggesting the evolutionary origin of Apd5 putative isomerases in PBD biosynthesis^36^.

In summary, limazepines are the only PBDs with a BGC encoding sequential homologues of all four above mentioned phenazine biosynthetic enzymes (Fig. 5), which indicates direct evolutionary links between the biosynthesis of limazepines and phenazines and which also provides an idea of the common origin of Apd5/PhzF isomerase activities.

## Conclusion

In addition to the already described kynurenine and chorismate/anthranilate pathways, we present the chorismate/DHHA pathway as the third natural concept employed to produce anthranilic acid derivatives as PBD precursors. Its elucidation raised the question whether this new way of biosynthesis of 3-hydroxyanthranilic acid is specific to limazepines or whether it is more general. Comparison of limazepine BGC to that of tilivalline/tilimycin (*lim6*/*adsX*, *lim5*/*icmX*, *lim4/dhbX*)^42^ shows that this PBD also employs the chorismate/DHHA pathway in its biosynthesis. Furthermore, genes homologous to *lim3-6*, encoding an enzyme of the shikimate pathway and enzymes of the chorismate/DHHA pathway, were identified within the BGCs of several other natural products structurally distinct from PBDs. These include paulomycins (*pau18-21*), diazepinomycin (*orf33,19,27,26*), or benzoxazoles such as calcimycin (*calB1-B4)*, caboxamycin (*cbxF-I*) or A33853 (*bomO-R*)^43–47^. Remarkably, part of the chorismate/DHHA pathway is shared also with phenazines. Unlike to all the previously listed metabolites, biosynthesis of phenazines has an additional evolutionary linkage with PBD biosynthesis: a common evolutionary origin of PhzF and Apd5 isomerases. Consequently, we propose that the here reported limazepine BGC may represent an important record of events in the evolution of phenazines and PBDs with an APD moiety.

## Electronic supplementary material

Supplementary material
