# Supplementary material for: Novel pathway of 3-hydroxyanthranilic acid formation in limazepine biosynthesis reveals evolutionary relation between phenazines and pyrrolobenzodiazepines
Source: Sci Rep. 2018 May 17;8:7810. doi: 10.1038/s41598-018-26179-w (PMC5958127; doi:10.1038/s41598-018-26179-w)
Supplement: Supplementary file 1 — Supplementary material [file 41598_2018_26179_MOESM1_ESM.docx]

**Novel pathway of 3-hydroxyanthranilic acid formation in limazepine biosynthesis reveals evolutionary relation between phenazines and pyrrolobenzodiazepines**

Magdalena Pavlikova, Zdenek Kamenik, Jiri Janata, Stanislav Kadlcik, Marek Kuzma, Lucie Najmanova*

Institute of Microbiology of the Czech Academy of Sciences, 142 20 Prague 4, Czech Republic

**Content:**

**Supplementary Figure 1A, 1B, 1C** page 2-4

**Supplementary Table 1** page 5

**Supplementary Table 2** page 6

**Supplementary Figure 2** page 7

**Supplementary Figure 3** page 8

**References** page 9

**Supplementary Figure 1**

**A) Sequence alignment of Lim5, PhzD and homologous proteins from putative chorismate/DHHA pathways from biosyntheses of various secondary metabolites.**

The alignment was generated using MAFFT web tool version 7.380. The identical residues are highlighted in rectangles. CalB2 - *Streptomyces chartreusis* NRRL 3882 (GenBank No: AEH42480.1), Orf27 - *Micromonospora* sp. M42 (GenBank No: EWM63057.1), BomP - *Streptomyces* sp. NRRL 12068 (GenBank No: ALE27508.1), CbxH - *Streptomyces* sp. NTK 937 (GenBank No: KDQ70112.1), Pau19 - *Streptomyces paulus* NRRL8115 (GenBank No: AIE54186.1), PhzD - *Pseudomonas fluorescens* 2-79 NRRL B-15132 (GenBank No: AAC18903.1).

**
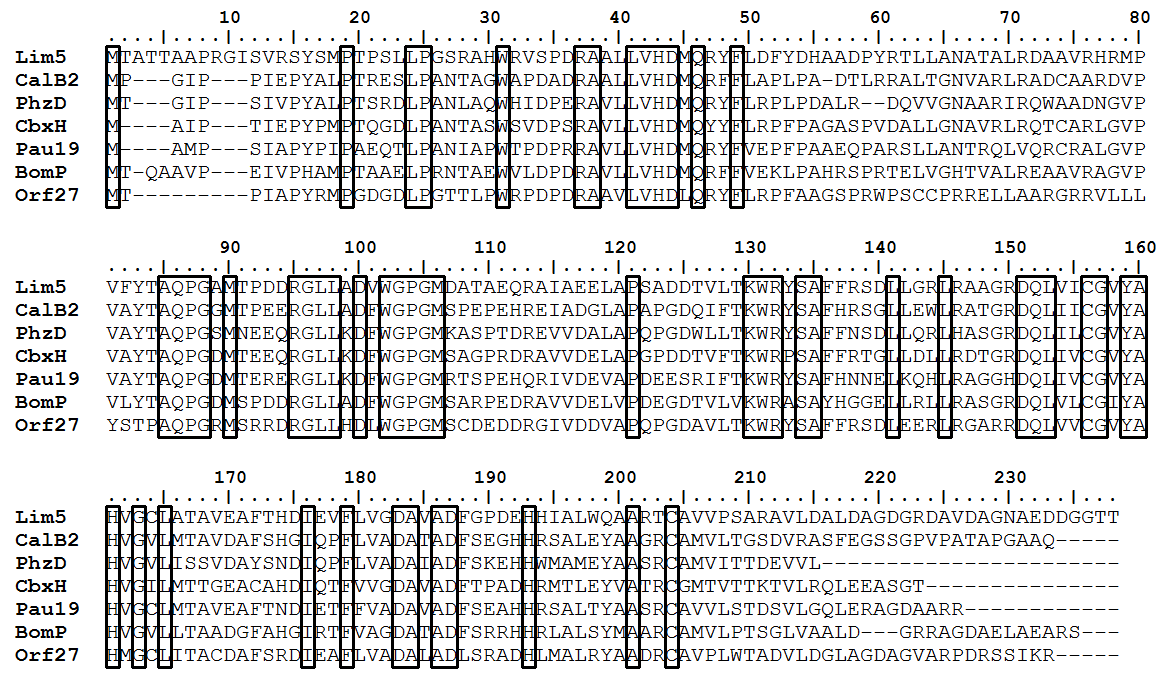
**

**B) Sequence alignment of Lim6, PhzE and homologous proteins from putative chorismate/DHHA pathways from biosyntheses of various secondary metabolites.**

The alignment was generated using MAFFT web tool version 7.380. The identical residues are highlighted in rectangles. The original CalB1 sequence in the GenBank is not correct since it starts from an internal methionine codon i.e. it is shortened at N-terminus. We analysed the genome data of *Streptomyces chartreusis* and corrected the CalB1 sequence and added it into the our alignment. The position of the previously presumed N-terminal methionine in CalB1 is marked with red asterisk. Also the original BomQ sequence in the GenBank is not correct since the presumed stop codon is probably result of a sequencing error. The sequence of BomQ continue further as can be seen from our alignment. The last residue (glycine) of the original incorrect BomQ sequence is marked with red triangle. However, an internal error in the published sequence did not allow to infer whether the sequence of BomQ is now correct or an amino acid residue/residues is still missing in the place of the sequencing error. CalB1 - *Streptomyces chartreusis* NRRL 3882 (GenBank No: AEH42479.1), Orf19 - *Micromonospora* sp*.* M42 (GenBank No: EWM63049.1), BomQ - *Streptomyces* sp. NRRL 12068 (GenBank No: ALE27509.1), CbxI - *Streptomyces* sp*.* NTK 937 (GenBank No: KDQ70113.1), Pau18 - *Streptomyces paulus* NRRL8115 (GenBank No: AIE54185.1), PhzE - *Pseudomonas fluorescens* 2-79 NRRL B-15132 (GenBank No: AAC18904.1).

**
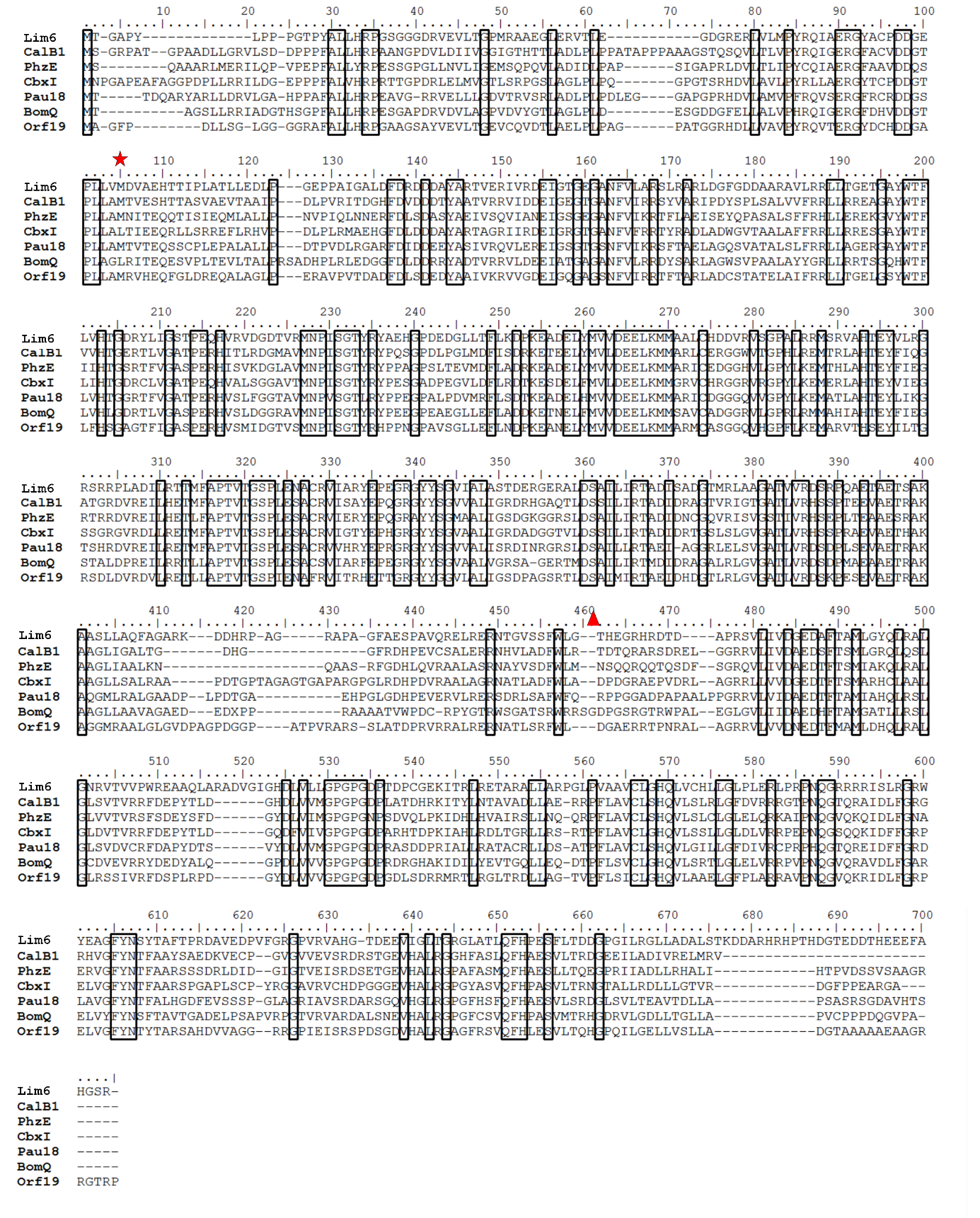
**

**C) Sequence alignment of proline racemase and representatives of homologous proteins from phenazine biosyntheses and APD pathways.**

The alignment was generated using MAFFT web tool version 7.380. The identical residues are highlighted in rectangles. Positions of cysteine residues in active site of proline racemase are marked with red asterisks, position of catalytic glutamate of PhzF conserved also in Lim13 and its homologs (Apd5) is highlighted in color and marked with yellow asterisk. proline racemase - *Clostridioides difficile* (GenBank No: ABS82398.1); Phenazine biosynthesis: PhzF - *Pseudomonas fluorescens* 2-79 NRRL B-15132 (GenBank No: AAC18905.1), PpzF - *Streptomyces anulatus* (GenBank No: CAX48668.1), EsmA3 - *Streptomyces antibioticus* Tu 2706 (GenBank No: AFB35622.1), LphzF - *Streptomyces lomondensis* (GenBank No: AKC91616.1), PhzF - *Lysobacter antibioticus* OH13 (GenBank No: AMQ09365.1); APD biosynthesis: Orf15 - *Streptomyces refuineus* (GenBank No: ABW71846.1), Por16 - *Streptomyces albus* subsp. *albus* (GenBank No: AEA29639.2), SibS - *Streptosporangium sibiricum* (GenBank No: ACN39743.1), TomK - *Streptomyces achromogenes* (GenBank No: ACN39024.1), LmbX - *Streptomyces lincolnensis* (GenBank No: ABX00621.1).

**
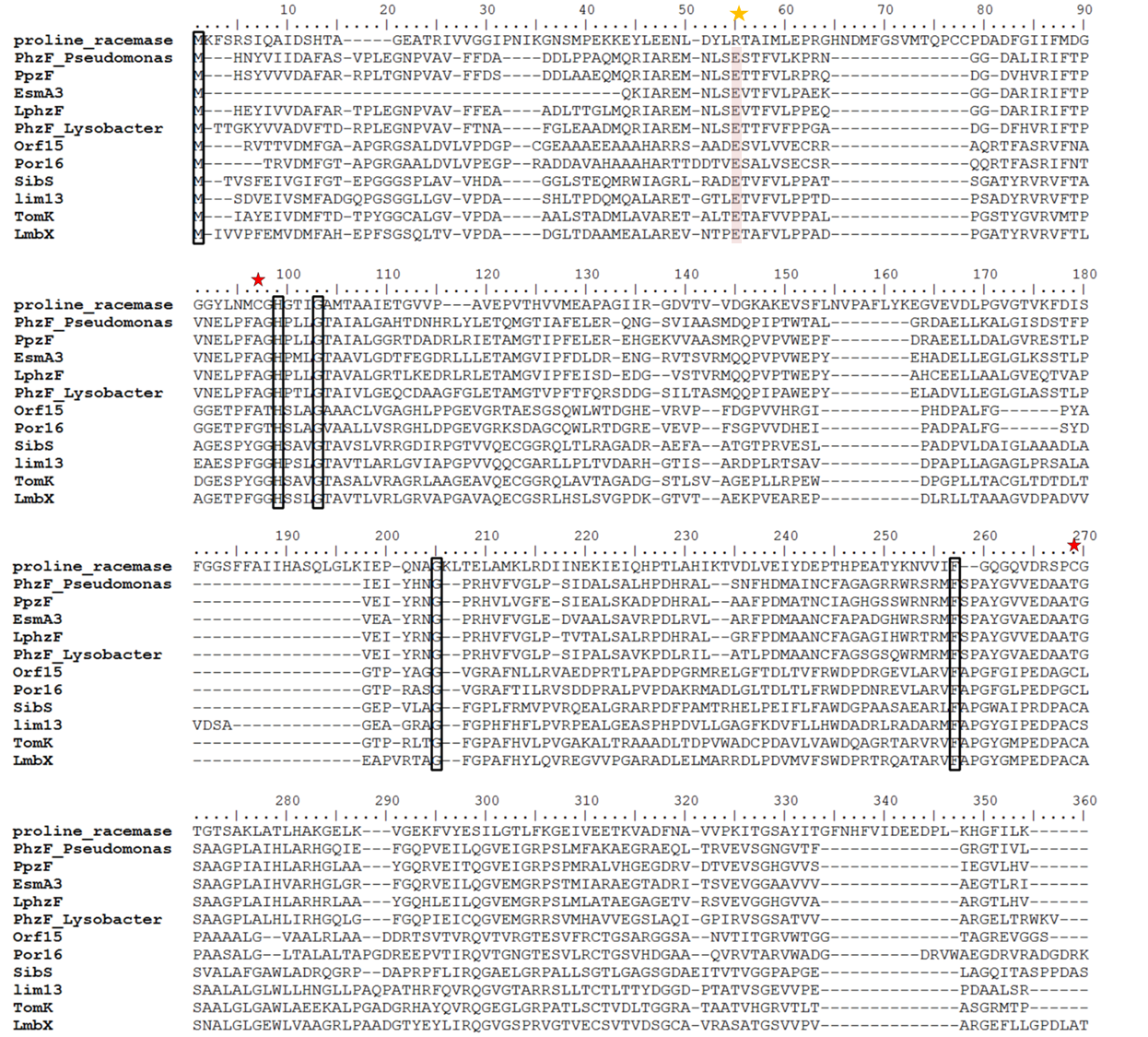
**

**Supplementary Table 1**

**NMR data of ADIC**

| **Atom #** | **δ_C_** | **m.** | **δ_H_** | **m.** | ***J*_HH_ [Hz]** |
| --- | --- | --- | --- | --- | --- |
| **1** | 77.25 | d | 4.987 | ddd | 1.7, 2.8, 10.8 |
| **2** | 54.33 | d | 4.512 | dd | 2.0, 10.8 |
| **3** | 131.99 | s | - |  |  |
| **4** | 134.76 | d | 6.942 | ddd | 1.2, 2.0, 5.4 |
| **5** | 128.62 | d | 6.305 | ddd | 1.7, 5.4, 9.8 |
| **6** | 130.79 | d | 6.267 | ddd | 1.2, 2.8, 9.8 |
| **1’** | 173.01 | s | - |  |  |
| **2’** | 155.62 | s | - |  |  |
| **3’** | 97.57 | t | 5.279 | d | 3.0 |
|  |  |  | 4.651 | d | 3.0 |
| **3-COOH** | 175.39 | s | - |  |  |

**Instrument:** Bruker Avance III 700 MHz (700.13 MHz for ^1^H, 176.05 MHz for ^13^C, 70.94 MHz for ^15^N, D_2_O, 30 ^o^C)

**Experiments performed:** ^1^H NMR,  ^13^C NMR, gCOSY, ^1^H-^13^C gHSQC, ^1^H-^13^C gHMBC

**Referencing:** ^1^H NMR and ^13^C NMR spectra were referenced using the residual signal of water (δ_H_ 4.736 ppm) and acetone (δ_C_ 33.08 ppm)

**Supplementary Table 2**

**NMR data of DHHA**

| **Atom #** | **δ_C_** | **m.** | **δ_H_** | **m.** | ***J*_HH_ [Hz]** |
| --- | --- | --- | --- | --- | --- |
| **1** | 68.61 | d | 4.328 | ddd | 1.0, 4.5, 5.2 |
| **2** | 54.20 | d | 4.132 | dd | 0.9, 5.2 |
| **3** | 131.21 | s | - |  |  |
| **4** | 135.05 | d | 6.901 | ddd | 0.9, 0.9, 5.7 |
| **5** | 128.31 | d | 6.234 | ddd | 1.0, 5.7, 9.5 |
| **6** | 132.81 | d | 6.110 | ddd | 0.9, 4.5, 9.6 |
| **3-COOH** | 175.69 | s | - |  |  |

The relative stereochemistry of DHHA was not independently assigned. The OH and NH_2_ group can exist as diaxial or diequatorial conformers as it was also reported for ADIC^1^. But the set of coupling constants is close to that one reported by McDonald et al^2^.

**Instrument:** Bruker Avance III 600 MHz (600.13 MHz for ^1^H, 150.95 MHz for ^13^C, 60.82 MHz for ^15^N, D_2_O, 15 ^o^C)

**Experiments performed:** ^1^H NMR,  ^13^C NMR, gCOSY, ^1^H-^13^C gHSQC, ^1^H-^13^C gHMBC

**Referencing:** ^1^H NMR spectrum was referenced using the residual signal of water (δ_H_ 4.843 ppm) and ^13^C NMR was referenced using acetone as an external reference (δ_C_ 30.09 ppm).

**Supplementary Figure 2**

**Ion-extracted LC-MS chromatograms of enzymatic reactions with Lim6.**

Positive control – conversion of chorismic acid to ADIC by Lim6 (ADIC was detected as a reaction product); negative control – no Lim6, *trans*-3,4-dihydroxybenzoic acid as a substrate; reaction – unsuccessful conversion of *trans*-3,4-dihydroxybenzoic acid to DHHA by Lim6 (no reaction product was detected). Lim 6 is capable of processing only chorismic acid, not *trans*-3,4-dihydroxybenzoic acid.

**
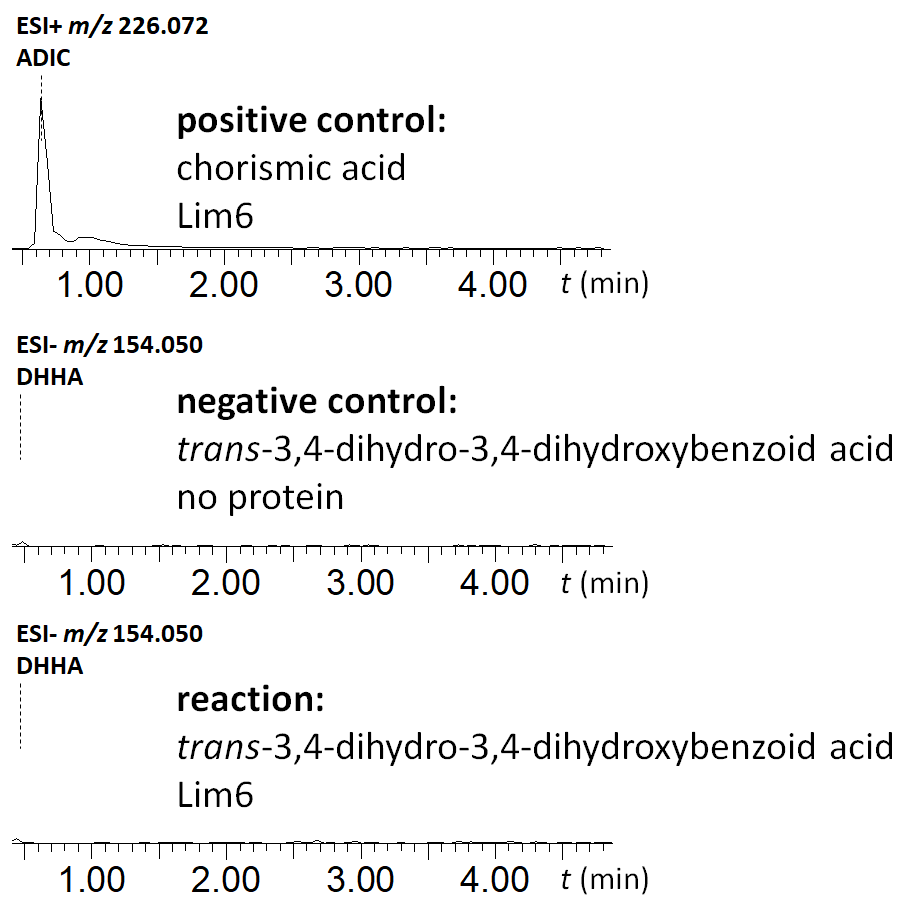
**

**Supplementary Figure 3**

**Rooted maximum-likelihood phylogenetic tree of Lim13 homologs.**

Multiple sequence alignment was generated using MAFFT web tool (version 7.380) and the tree was constructed using MEGA7^3^. Bootstrap values (100 replicates) are indicated at the nodes. Proline racemase from *Clostridioides difficile* (GenBank No: ABS82398.1) was used as the outgroup. Phenazine biosynthetic proteins: PhzF - *Pseudomonas fluorescens* 2-79 NRRL B-15132 (GenBank No: AAC18905.1), PpzF - *Streptomyces anulatus* (GenBank No: CAX48668.1), EsmA3 - *Streptomyces antibioticus* Tu 2706 (GenBank No: AFB35622.1), LphzF - *Streptomyces lomondensis* (GenBank No: AKC91616.1), PhzF - *Lysobacter antibioticus* OH13 (GenBank No: AMQ09365.1), PhzF - *Pseudomonas chlororaphis* G05 (GenBank No: AME16515.1), PhzF - *Pseudomonas aeruginosa* PAO1 (GenBank No: AAC64492.1), EpzF - *Streptomyces cinnamonensis* (GenBank No: ADQ43387.1), Cnq727 - *Streptomyces* sp. CNQ-509 (GenBank No: AIT42117.1); PBD biosynthesis: Orf15 - *Streptomyces refuineus* (GenBank No: ABW71846.1), Por16 - *Streptomyces albus* subsp. *albus* (GenBank No: AEA29639.2), SibS - *Streptosporangium sibiricum* (GenBank No: ACN39743.1), TomK *- Streptomyces achromogenes* (GenBank No: ACN39024.1), LmbX - *Streptomyces lincolnensis* (GenBank No: ABX00621.1); *Streptomyces purpureus* (WP_040875305.1), *Actinomadura echinospora* (SEG83576.1), *Streptomyces* sp. CNS654 (WP_032765882.1), *Streptomyces scabrisporus* (WP_020555669.1), *Streptomyces* sp. NRRL WC-3742 (WP_031068412.1), *Streptomyces sclerotialus* (WP_037773338.1), *Micromonospora echinospora* (SCF40027.1), *Dermacoccus* sp. PE3 (WP_052947175.1), *Nocardiopsis prasina* (WP_017545370.1)- see review^4^.

**
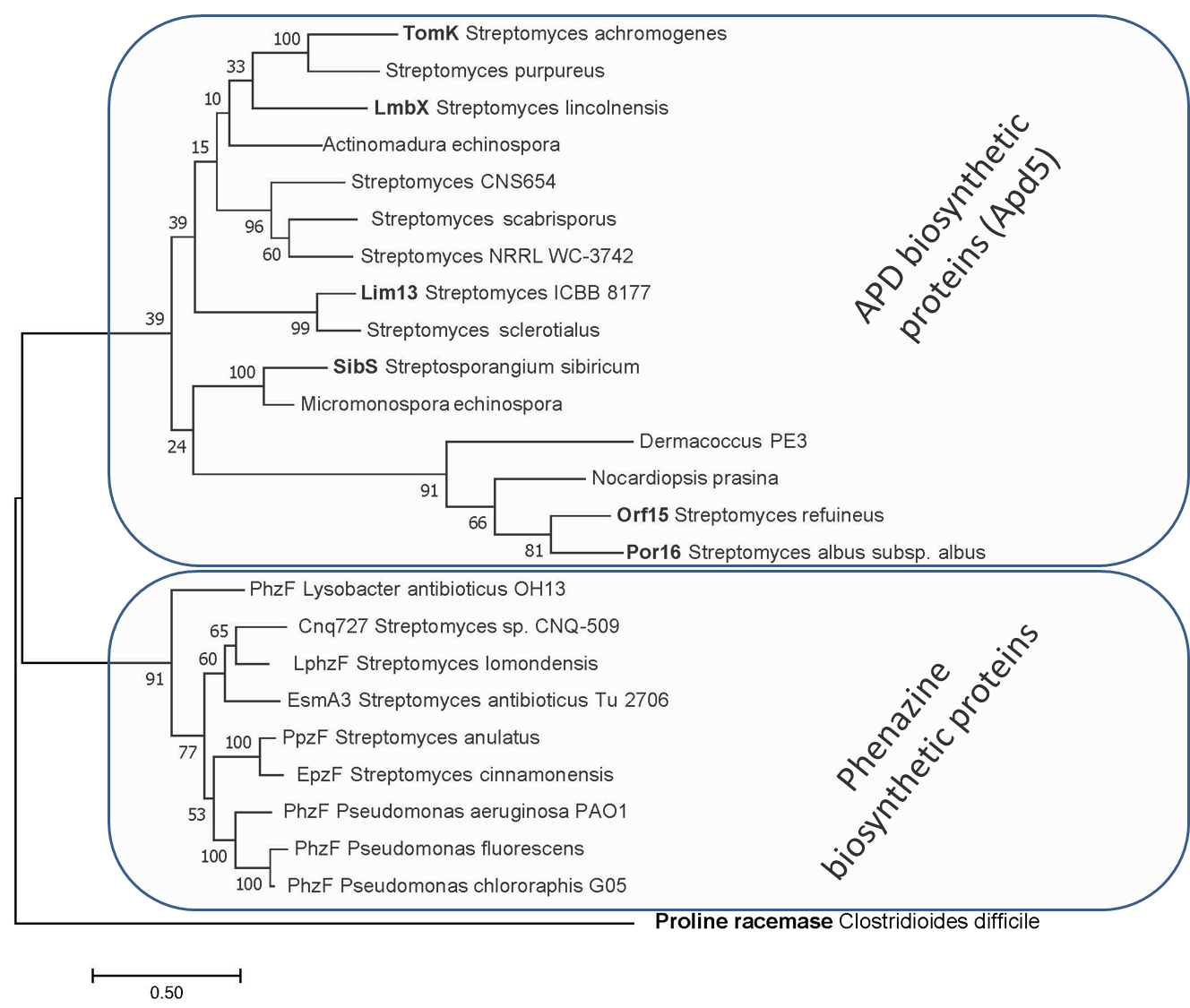
**

**References:**

1. Morollo, A. A., Finn, M. G. & Bauerle, R. Isolation and structure determination of 2-amino-2-deoxyisochorismate: an intermediate in the biosynthesis of anthranilate. *J. Am. Chem. Soc.* **115,** 816–817 (1993).

2. McDonald, M., Mavrodi, D. V, Thomashow, L. S. & Floss, H. G. Phenazine biosynthesis in pseudomonas fluorescens: branchpoint from the primary shikimate biosynthetic pathway and role of phenazine-1,6-dicarboxylic acid. *J. Am. Chem. Soc.* **123,** 9459–9460 (2001).

3. Kumar, S., Stecher, G. & Tamura, K. MEGA7: Molecular evolutionary genetics analysis version 7.0 for bigger datasets. *Mol. Biol. Evol.* **33,** 1870–1874 (2016).

4. Janata, J., Kamenik, Z., Gazak, R., Kadlcik, S. & Najmanova, L. Biosynthesis and incorporation of an alkylproline-derivative (APD) precursor into complex natural products. *Nat. Prod. Rep.* **35,** 257–289 (2018).
